# Supplementary material for: Content-rich biological network constructed by mining PubMed abstracts
Source: BMC Bioinformatics. 2004 Oct 8;5:147. doi: 10.1186/1471-2105-5-147 (PMC528731; doi:10.1186/1471-2105-5-147)
Supplement: Additional File 5 — The original Chilibot query results of the term "long-term potentiation (LTP)" and 22 other terms, limiting the latest references analyzed to the years 1990, 1995, 2000, and 2004. [file 1471-2105-5-147-S5.bz2 › chilibotAdditionalFile5/ltp2004/html/LTP_ERK.html]

 


 **LTP** and **ERK** 
  
Found 33 abstracts in PubMed,  **30 abstracts were retrieved and analyzed**.  


---

 Search Google  |
 PDF files only 
|  EDU domain only 

---

**Interactive relationship** (e.g. stimulation, inhibition, etc)

- However, in mice, inhibition of  **ERK**  activation blocked  **LTP**  induced by two  **LTP**  induction paradigms that mimicked the endogenous theta rhythm.  Ref: 12551961 Learn MemLearn Mem, 1997
- **ERK**  plays a regulatory role in induction of  **LTP**  by theta frequency stimulation and its modulation by beta adrenergic receptors.  Ref: 10595521 Neuron, 1999
- Activation of MEK  **ERK**  is therefore required for the induction... of BDNF  **LTP** .  Ref: 11880483 J Neurosci, 2002
- Together, our results indicate that the role of PI3 kinase in  **LTP**  is not limited to its role as an upstream regulator of MAPK signaling but also includes signaling through  **ERK**  independent pathways that regulate  **LTP**  induction.  Ref: 12736339 J Neurosci, 2003
- We also show that inhibition of the MAPK  **ERK**  cascade by the MAPK kinase MEK inhibitor SL327 prevented CREB and Elk 1 phosphorylation, and  **LTP**  dependent gene induction, resulting in rapidly decaying  **LTP** .  Ref: 10844026 J Neurosci, 2000
- We found that  **ERK**  was activated in area CA1 after the induction of both 200 Hz  **LTP**  and TEA  **LTP** .  Ref: 10777769 J Neurosci, 2000
- Activation of extracellular signal regulated kinase  **ERK**  has been shown to be necessary for NMDA receptor dependent long term potentiation  [ **LTP** ]   **LTP** .  Ref: 10777769 J Neurosci, 2000
- By examining the role of FAK involved in  **LTP**  induction in dentate gyrus in vivo with medial perforant path stimulation, we found that both FAK and mitogen activated protein kinase MAPK extracellular signal regulated kinase  **ERK**  phosphorylation were increased significantly 5 and 10 min after  **LTP**  induction.  Ref: 12764094 J Neurosci, 2003
- **ERK**  is required for the early phase of  **LTP**  elicited by brief 5 Hz stimulation, as well as for  **LTP**  elicited by more prolonged 5 Hz stimulation when paired with beta1 adrenergic receptor activation.  Ref: 10595521 Neuron, 1999
- Here we show that induction of long term potentiation  [ **LTP** ]   **LTP**  in the dentate gyrus in vivo leads to rapid phosphorylation and nuclear translocation of MAPK  **ERK** .  Ref: 10844026 J Neurosci, 2000
- **ERK**  activation has been shown to be required for the induction of long term potentiation  [ **LTP** ]   **LTP**  in the rat hippocampus and for the formation of associative and spatial memories in both the rat and the mouse.  Ref: 11160759 Learn MemLearn Mem, 2000
- Long term potentiation  [ **LTP** ]  and spatial learning are associated with increased phosphorylation of TrkB and extracellular signal regulated kinase  **ERK**  in the dentate gyrus evidence for a role for brain derived neurotrophic factor.  Ref: 12049326 Behav Neurosci, 2002
- We have found that  **ERK**  also regulates hippocampal neuronal excitability during 5 Hz stimulation and thereby regulates forms of long term potentiation  [ **LTP** ]   **LTP**  that do not require macromolecular synthesis.  Ref: 10595521 Neuron, 1999
- Overall, our observations indicate that activation of the  **ERK**  signaling cascade is necessary for NMDA receptor independent  **LTP**  in area CA1 but not in area CA3 and suggest a divergence in the signaling cascades underlying NMDA receptor independent  **LTP**  in these hippocampal subregions.  Ref: 10777769 J Neurosci, 2000
- BDNF  **LTP**  was further coupled to  **ERK**  dependent phosphorylation of the transcription factor cAMP response element binding protein.  Ref: 11880483 J Neurosci, 2002
- By contrast,  **ERK**  plays no role in  **LTP**  elicited by a single 1 s 100 Hz train.  Ref: 10595521 Neuron, 1999
- The NO cGMP PKG  **ERK**  signaling pathway thus plays important role in the potentiation of  **LTP**  by YC 1.  Ref: 12761342 Mol Pharmacol, 2003
- These results demonstrate that the  **ERK**  pathway is necessary for experience dependent plasticity and for  **LTP**  of synaptic transmission in the developing visual cortex.  Ref: 11423664 Science, 2001
- Moreover,  **ERK**  mediated changes in excitability are selectively required for some forms of  **LTP**  but not others.  Ref: 10595521 Neuron, 1999
- Our findings indicate that the  **ERK**  cascade transduces signals from the synapse to the nucleus during LTD in hippocampal area CA1 in vivo, as it does during long term potentiation  [ **LTP** ]  in area CA1, but that the pattern of coupling of the  **ERK**  cascade to transcriptional regulators differs between the two forms of synaptic plasticity.  Ref: 11896145 J Neurosci, 2002
- Here, we briefly review experiments that have shown MAPK  **ERK** , cAMP response element binding protein CREB and the immediate early gene IEG zif268 are essential components of a signalling cascade required for the expression of late phase  **LTP**  and of certain forms of long term memory.  Ref: 12740127 Philos Trans R Soc Lond B Biol Sci, 2003
- We show that two different inhibitors of the  **ERK**  pathway suppress the induction of two forms of long term potentiation  [ **LTP** ]   **LTP**  in rat cortical slices.  Ref: 11423664 Science, 2001
- Inhibition of the  **ERK**  signaling cascade with either PD 098059 or U0126 prevented the induction of both 200 Hz  **LTP**  and TEA  **LTP**  in area CA1.  Ref: 10777769 J Neurosci, 2000

**Parallel relationship** (e.g. studied together, co-existance, homology, etc.)

- Consistent with this, the amount of potentiation induced by stimulation protocols that induce an  **ERK**  independent form of  **LTP**  were also significantly reduced in slices from SynGAP mice.  Ref: 12427827 J Neurosci, 2002
- **LTP**  was also associated with an increase in phosphorylation of the alpha subunit of calcium channels and  **ERK**  in synaptosomes prepared from dentate gyrus, and these effects were inhibited when  **LTP**  was blocked by the tyrosine kinase inhibitor, genistein.  Ref: 12099488 Hippocampus, 2002
- Because activation of the extracellular signal regulated kinase  **ERK**  has been shown to be necessary for long term potentiation  [ **LTP** ] , we investigated the regulation of  **ERK**  in long term depression LTD in the adult hippocampus in vivo.  Ref: 10617120 J Neurochem, 2000
- Moreover, blockade of NMDA receptor NMDAR did not decrease FAK, MAPK  **ERK** , and CREB phosphorylation although  **LTP**  induction was blunted by NMDAR antagonist.  Ref: 12764094 J Neurosci, 2003
- Given the possible role of altered gene expression in the late phase of  **LTP**  and in long term memory, we evaluated the capacity of the MAPK  **ERK**  extracellular signal regulated kinase to regulate phosphorylation of the transcription factor cAMP response element binding protein CREB in hippocampal area CA1.  Ref: 11016081 Acta Neurobiol Exp (Wars), 2000
- Local infusion of MEK inhibitors during BDNF delivery abolished BDNF  **LTP**  and the associated  **ERK**  activation.  Ref: 11880483 J Neurosci, 2002
- PI3 kinase inhibitors also block activation of  **ERK**  by NMDA receptor stimulation, suggesting that PI3 kinase inhibitors block  **LTP**  because PI3 kinase is an essential upstream regulator of  **ERK**  activation.  Ref: 12736339 J Neurosci, 2003
- For example, the mitogen activated protein kinase, or extracellular signal regulated kinase  **ERK** , signaling cascade has increasingly been shown to be important for the induction of gene expression and long term potentiation  [ **LTP** ] .  Ref: 10652372 J Biol Chem, 2000
- Inhibitors of both phosphatidylinositol 3 kinase PI3 kinase and MAPK  **ERK**  mitogen activate protein kinase extracellular signal related kinase activation inhibit NMDA receptor dependent long term potentiation  [ **LTP** ]   **LTP** .  Ref: 12736339 J Neurosci, 2003
- RAS signaling is also known to mediate long term potentiation  [ **LTP** ]   **LTP**  and long term memory formation in postmitotic neurons, in part through activation of the RAF MEK  **ERK**  pathway.  Ref: 12574403 J Neurosci, 2003
- To investigate  **ERK**  dependent mechanisms operating during  **LTP**  inducing stimulation paradigms, we monitored spike production in the cell body layer of the hippocampus during the period of theta like  **LTP**  inducing stimulation.  Ref: 12551961 Learn MemLearn Mem, 2003
- Thus, MAPK  **ERK**  activation is required for  **LTP**  dependent transcriptional regulation and we suggest this is regulated by two parallel signaling pathways, the MAPK  **ERK**  Elk 1 pathway targeting SRE and the MAPK  **ERK**  CREB pathway targeting CRE.  Ref: 10844026 J Neurosci, 2000
- We studied the role of  **ERK**  in three forms of NMDA receptor independent  **LTP**   **LTP**  induced by very high frequency stimulation 200 Hz  **LTP** ,  **LTP**  induced by the K channel blocker tetraethylammonium TEA TEA  **LTP** , and mossy fiber MF  **LTP**  MF  **LTP** .  Ref: 10777769 J Neurosci, 2000
- To examine this hypothesis, we investigated the effects of PI3 kinase inhibitors on  **ERK**  activation and  **LTP**  induction in the CA1 region of mouse hippocampal slices.  Ref: 12736339 J Neurosci, 2003
- The phosphorylation state of a transcriptional mediator of long term potentiation  [ **LTP** ]  and a downstream target of the  **ERK**  MAPK cascade, the cAMP regulatory element binding protein, were affected also.  Ref: 11404397 J Neurosci, 2001
- We have studied the effects of interleukin 6 IL 6 on the expression of paired pulse facilitation PPF, posttetanic potentiation PTP, and long term potentiation  [ **LTP** ]   **LTP**  in the CA1 region of the hippocampus as well as on the activation of the signal transducer and activator of transcription 3 STAT3, the mitogen activated protein kinase  **ERK**  MAPK  **ERK** , and the stress activated protein kinase c Jun NH 2 terminal kinase SAPK JNK.  Ref: 10899938 J Neurochem, 2000
- The IL 6 induced inhibition of PTP and  **LTP**  was accompanied by a simulation of STAT3 tyrosine phosphorylation and an inhibition of MAPK  **ERK**  dual phosphorylation, in the absence of changes in the state of activation of SAPK JNK.  Ref: 10899938 J Neurochem, 2000
- We found that long term potentiation  [ **LTP** ]   **LTP**  induced with a pair of 100 Hz tetani does not require  **ERK**  activation in mice whereas it does in rats.  Ref: 12551961 Learn MemLearn Mem, 2000
- Disruption of lipid rafts inhibited NRG induced activation of  **Erk**  and prevented NRG induced blockade of induction of long term potentiation  [ **LTP** ]  at hippocampal CA1 synapses.  Ref: 12716924 J Neurosci, 2003
- The MAPK  **ERK**  cascade targets both Elk 1 and cAMP response element binding protein to control long term potentiation  [ **LTP** ]  dependent gene expression in the dentate gyrus in vivo.  Ref: 10844026 J Neurosci, 2000
- Thus, although SynGAP may regulate the  **ERK**  pathway, its role in  **LTP**  most likely involves additional downstream targets.  Ref: 12427827 J Neurosci, 2002
